# EEG-based hypoglycemia detection in Type 1 Diabetes: Proof-of-concept study

**DOI:** 10.3389/fnhum.2026.1852403

**Published:** 2026-08-13

**Authors:** Michal Kubaščík, Swati Aggarwal, Ondrej Karpiš, Andrej Tupý, Peter Šarafín, Miroslav Chochul

**Affiliations:** 1 Department of Technical Cybernetics, Faculty of Management Science and Informatics, University of Žilina, Žilina, Slovakia; 2 Faculty of Logistics, Molde University College, Molde, Møre og Romsdal county, Norway

**Keywords:** brain-computer interface, diabetes management, electroencephalography, machine learning, signal processing

## Abstract

Early detection of hypoglycemia among non-hypoglycemic conditions is critical in type 1 diabetes (T1D), as delayed intervention can lead to serious neurological and metabolic consequences. Although continuous glucose monitoring (CGM) systems provide continuous glucose measurements, they are invasive and do not capture early neurophysiological alterations associated with hypoglycemia-related metabolic changes. This study presents a preliminary feasibility investigation of a non-invasive brain–computer interface approach for classifying hypoglycemia versus non-hypoglycemia in individuals with type 1 diabetes using electroencephalography (EEG). The novelty lies in demonstrating EEG-based hypoglycemia detection (binary classification) under data-limited conditions using optimized segmentation and lightweight classifiers. Additionally, EEG characteristics were compared with recordings from five healthy controls to provide a baseline reference for spectral activity patterns. EEG recordings from participants with T1D were synchronized with continuous glucose monitoring (CGM) data and pre-processed using a 0.5-50 Hz band-pass filter. The control group underwent identical EEG pre-processing without CGM synchronization. Reproducible spectral patterns were identified: hypoglycemia was associated with characteristic delta and beta alterations, while changes observed outside hypoglycemia were less consistent and did not support reliable separation within the non-hypoglycemic class. Multiple segmentation strategies and data-efficient machine learning models were evaluated under limited data conditions. Classical classifiers demonstrated promising within-subject performance under severely data-limited conditions, with Quadratic Discriminant Analysis (QDA) achieving the best results (accuracy 0.96212, macro-F1 0.96201) for binary classification focused on hypoglycemia detection. Confusion matrix analysis indicated a low rate of clinically relevant misclassifications. These findings support the feasibility of lightweight, real-time, non-invasive EEG-based systems for early hypoglycemia detection.

## 1 Introduction

In healthy individuals, blood-glucose levels (BGL) are maintained within a narrow physiological range through the action of the hormone insulin, which facilitates cellular glucose uptake and its subsequent utilization for energy. When this regulatory mechanism fails, the body is unable to sustain normal glucose homeostasis, leading predominantly to chronic elevation of BGL. According to the National Institute of Diabetes and Digestive and Kidney Diseases, National Institute of Health, USA, diabetes mellitus encompasses a group of metabolic disorders characterized by impaired insulin function, either due to insufficient insulin production or reduced cellular responsiveness.

Type 1 diabetes (T1D) arises primarily from autoimmune-mediated destruction of pancreatic β-cells, resulting in minimal or absent endogenous insulin secretion. In contrast, type 2 diabetes (T2D) is associated with preserved insulin production but reduced insulin sensitivity, leading to ineffective glucose absorption by peripheral tissues. Additional forms include gestational diabetes, which develops during pregnancy, and prediabetes, defined by glucose levels that exceed normal values but do not meet the diagnostic criteria for T2D.

Diabetes is a chronic condition that affects millions of people worldwide and is frequently accompanied by critical events such as hypoglycemia and hyperglycemia. These events can lead to severe health complications, including cognitive impairment, cardiovascular issues, seizures, and, in extreme cases, death (Ngo et al., 2021). Early prediction of these critical events is essential to mitigating these risks and improving the quality of life for patients with diabetes.

In individuals with diabetes, glycemic states are typically categorized as euglycemia, defined by 70 < BGL < 180 mg/dL. Hypoglycemia, or low blood glucose (BGL < 70 mg/dL), is a major concern for individuals undergoing insulin treatment for T1D. Early symptoms include anxiety, sweating, and hunger; however, if untreated, hypoglycemia can progress to severe neurological outcomes such as seizures and loss of consciousness. Nocturnal hypoglycemia is particularly dangerous, as symptoms may go unnoticed during sleep, and in extreme cases may lead to the “dead-in-bed syndrome” (Ngo et al., 2021). Hyperglycemia (BGL>180 mg/dL) refers to elevated blood glucose levels and is associated with an increased risk of cardiovascular disease, vision impairment, and cognitive dysfunction. In patients with T1D, prolonged hyperglycemia may progress to diabetic ketoacidosis, a life-threatening metabolic emergency (Ngo et al., 2021).

In the context of bioelectronic and metabolic monitoring, critical events are defined as life-threatening emergencies or pathological activities that arise when a physiological parameter deviates from its stable state, necessitating prompt clinical recognition and protocol-based intervention (Patel, 2025; Gunduz et al., 2019). These events are formally characterized by risk, defined as the combination of the probability of occurrence of harm and the severity of that harm, where harm refers to physical injury or damage to human health (Gunduz et al., 2019). Within “smart” bioelectronic frameworks, a critical event occurs when a system fails to maintain a desired physiological set-point, potentially resulting in hazards such as under- or overstimulation that place the patient at immediate risk (Gunduz et al., 2019).

Such events often manifest episodically as symptoms of underlying neurological or metabolic disorders, including seizures, tremors, or comatose states, driven by erratic neural activity or significant metabolic excursions (Gunduz et al., 2019; Sindagi and Mahesh, 2019; EL-Mohandes et al., 2023). Because these occurrences may be infrequent or develop gradually over extended periods, they are categorized as glycemic or neurological emergencies that require automated sensing technologies capable of detecting physiological signals that may remain imperceptible to the patient (Dave et al., 2024; Sindagi and Mahesh, 2019; Gunduz et al., 2019; Patel, 2025). Ultimately, the definition of a critical event encompasses any acute physiological disturbance in which the brain's ability to sense or regulate essential parameters is impaired, leading to metabolic instability or systemic toxicity (James et al., 2021; Patel, 2025).

Effective management and prevention of critical glycemic events increasingly rely on advanced technological and lifestyle interventions. Continuous glucose monitoring (CGM) systems provide real-time feedback on glucose dynamics, enabling rapid detection of deviations. Automatic insulin delivery systems, often referred to as closed-loop systems, further enhance glycemic control by adjusting insulin administration based on CGM data and physiological parameters. Together, these technologies form the foundation of modern diabetes management and have significantly improved metabolic outcomes and patient safety.

Despite these advances, CGM-based systems remain invasive and may suffer from calibration drift, sensor latency, or failure, motivating the exploration of complementary monitoring modalities. Electroencephalography (EEG), a non-invasive method for measuring brain activity, offers a promising alternative. EEG measurements provide direct insight into neurophysiological processes that are affected by fluctuations in glucose levels. Emerging evidence suggests that critical diabetes-related events induce distinct patterns of neural activity that can be captured and analyzed using EEG signals (Ryu et al., 2019). Unlike traditional blood glucose monitoring methods, EEG has the potential to provide continuous, real-time information without requiring direct blood or interstitial fluid measurements.

The objective of this study is to develop a non-invasive approach for classifying critical diabetes-related events using EEG signals within a BCI framework. By applying advanced signal processing and machine learning techniques, this work aims to characterize the relationship between EEG-derived features and glycemic states. Although EEG-based detection of glycemic disturbances has been examined in previous research, there is a paucity of systematic investigations into how segmentation strategies and model complexity affect classification performance in data-limited BCI scenarios. Accordingly, this study focuses on the practical classification of critical glycemic events and provides a structured comparison of different segmentation strategies and machine learning models under realistic constraints on data availability. Particular emphasis is placed on computationally efficient and low-complexity models, motivated by the requirements of embedded and wearable BCI systems, where processing power, memory resources, energy consumption, and inference latency are strictly limited. A central contribution of this work is the empirical demonstration that simple, data-efficient classifiers can outperform deep learning-based approaches under limited training data conditions, while also offering advantages in computational complexity, real-time responsiveness, and deployment feasibility in embedded platforms. The following sections detail the methodology, datasets, analytical models, and experimental results that underpin this approach.

While glycemic states are conventionally categorized as hypoglycemia, euglycemia, and hyperglycemia, the present work focuses on binary classification of hypoglycemia versus non-hypoglycemia. This formulation reflects clinical priorities, where rapid detection of hypoglycemia is critical, and is further motivated by the limited separability between hyperglycemia and euglycemia observed in EEG features.

## 2 Literature review

### 2.1 Effect of hypoglycemia on brain activity

Several studies have reported cases in which individuals did not exhibit significant EEG changes during hypoglycemia, even in the presence of clear symptoms. One proposed explanation is the protective role of glucagon as a counter-regulatory mechanism against neuroglycopenia (Blaabjerg and Juhl, 2016). This hypothesis is supported by observations of absent hypoglycemia-induced EEG changes during both nocturnal and daytime hypoglycemia in patients with preserved glucagon responses. These individuals were also characterized by low basal renin–angiotensin activity, suggesting the involvement of additional mechanisms influencing cerebral signal processing.

However, other investigations have challenged the association between glucagon response and EEG alterations, reporting hypoglycemia-induced EEG changes regardless of counter-regulatory glucagon activity in both diabetic and healthy individuals. In subjects without detectable EEG changes, glucose nadir values ranged from 28.8 to 39.96 mg/dL, indicating substantial inter-individual variability. In some cases, insufficient hypoglycemia depth may explain the absence of EEG changes. Nevertheless, residual alpha activity and limited slow-wave activity in the 4—7 Hz range have been reported at extremely low glucose levels (below 14.94 mg/dL) in patients with epilepsy, although centroid frequency was not assessed and pre-existing EEG abnormalities may have masked hypoglycemia-related effects. Overall, EEG slowing during hypoglycemia appears to be a robust phenomenon across age groups, metabolic status, and hypoglycemia etiology (Blaabjerg and Juhl, 2016).

Under hyperinsulinemic hypoglycemia, cerebral responses have been quantified using spectral moments of EEG signals, including centroid frequency and spectral gyration radius. In a study of five adolescents with T1D monitored over 10 days, nocturnal hypoglycemic episodes were associated with significant spectral moment changes, most prominently in occipital regions. Effect size analysis identified 30-s EEG segments as optimal for hypoglycemia detection, and Bayesian neural networks achieved the highest detection performance, with Clarke's error grid analysis indicating 86% of estimated glucose values within clinically acceptable ranges (Ngo et al., 2021).

The influence of recurrent hypoglycemia on EEG responses has also been examined. In a cohort of 24 patients with T1D, classified as hypoglycemia-aware or unaware, EEG recordings obtained during two consecutive hypoglycemic clamp days revealed consistent hypoglycemia-associated EEG changes. No significant differences were observed in theta, alpha, or combined theta–alpha band power or frequency distributions between days or between awareness groups, indicating that antecedent hypoglycemia does not significantly alter hypoglycemia-related EEG changes (Sejling et al., 2017).

Further evidence from EEG and visual evoked potential recordings during hypoglycemic clamp studies demonstrated a significant reduction in mean and peak alpha-band frequency in frontal EEG leads during hypoglycemia, while visual evoked potential latencies remained unchanged between euglycemic and hypoglycemic states (Tamburrano et al., 1988). These findings suggest that EEG frequency measures are more sensitive indicators of hypoglycemia than visual evoked potentials.

Additional studies using EEG channels C3, C4, O1, and O2 reported significant increases in theta spectral moments and corresponding decreases in alpha moments during hypoglycemia. By prioritizing sensitivity over specificity, nocturnal hypoglycemia detection achieved 85% sensitivity and 74% accuracy, with 93% of estimated glucose values classified as clinically acceptable. The proposed spectral feature extraction algorithm exhibited linear computational complexity, supporting its suitability for real-time, non-invasive BCI applications (Ngo et al., 2021).

### 2.2 Effect of hyperglycemia on brain activity

Hyperglycemia-related EEG alterations have been investigated using continuous EEG and glucose monitoring in youths with T1D. In a 40-h study involving nine participants, EEG power across frequency bands from 0.5 to 80 Hz was analyzed relative to interstitial glucose concentration ranges of 72.0—191.0 mg/dL, 191.0—279.0 mg/dL, and above 279.0 mg/dL. The results demonstrated a non-linear relationship between EEG power and glucose concentration, with significant differences observed across all frequency bands during both wakefulness and sleep when elevated glucose levels were compared with euglycemia (Rachmiel et al., 2016).

During wakefulness, severe hyperglycemia was associated with reduced power in high-frequency bands (beta and gamma) and increased power in low-frequency bands (delta and theta), while moderate hyperglycemia showed increased high-frequency activity relative to euglycemia. During sleep, elevated glucose levels were linked to increased EEG power across most frequency bands, with stronger effects at higher glucose concentrations (Rachmiel et al., 2016).

Further analysis of EEG recordings from four T1D patients using 20-s segments aligned with blood sampling points confirmed non-linear changes in EEG band power with respect to blood glucose levels. The most pronounced differences were observed in the alpha band (electrodes C4, O1, and O2) and the beta band (electrodes C3, C4, O1, and O2), indicating both regional and frequency-specific sensitivity to hyperglycemia (Ngo et al., 2020).

Additional studies evaluating EEG spectral moments using a hyperglycemic threshold of 149.4 mg/dL reported significant correlations between hyperglycemia and alpha- and beta-band activity, with increased spectral moments during hyperglycemic episodes. These effects were more prominent in parietal and occipital regions than in central areas, further supporting the spatial specificity of hyperglycemia-induced EEG alterations (Ngo et al., 2021).

### 2.3 BCI-based classification of glucose-related physiological events

The classification of critical physiological events, including hypoglycemic episodes, seizures, and tremors, using BCI is typically based on a structured processing pipeline consisting of signal acquisition, artifact removal, feature extraction, and machine learning-based classification. This framework provides a methodological basis for translating glucose-related EEG alterations into automated detection systems.

BCI systems acquire neural signals through invasive approaches, such as implanted microelectrode arrays, or non-invasive modalities including scalp and ear-EEG (Satam and Szabolcsi, 2025; Cabrera et al., 2024; Gunduz et al., 2019). Non-invasive EEG is particularly suitable for long-term glucose monitoring; however, it is susceptible to artifacts caused by muscle activity, eye movements, and environmental noise. Consequently, adaptive filtering methods such as Least mean squares (LMS) and Recursive least squares (RLS), together with Independent Component Analysis (ICA), are commonly applied to improve signal quality (Sindagi and Mahesh, 2019).

After pre-processing, discriminative features are extracted to characterize physiological states. Spectral features derived from canonical EEG frequency bands are widely used, with hypoglycemia commonly associated with increased theta activity and reduced alpha frequency (Cabrera et al., 2024; Sindagi and Mahesh, 2019). Additional temporal and spatial features, including statistical moments and Common Spatial Patterns (CSP), further enhance class separability (Gunduz et al., 2019). Event-related potentials (ERP) have also been investigated as potential early indicators of glucose changes (Connor, 2017).

Classification is typically performed using traditional machine learning models such as Support Vector Machines (SVM), Linear Discriminant Analysis (LDA), and Random Forests (Hasan and Yasmin, 2025). More advanced hybrid architectures, including CNN–LSTM models such as DNet, have demonstrated high classification accuracy in diabetic risk prediction tasks (Hasan and Yasmin, 2025). In closed-loop bioelectronic systems, classifier outputs may be used to trigger real-time interventions, including stimulation-based therapies or automated insulin delivery in response to hypoglycemia-related EEG signatures (Gunduz et al., 2019; Connor, 2017).

## 3 Methods

**Figure 1:**
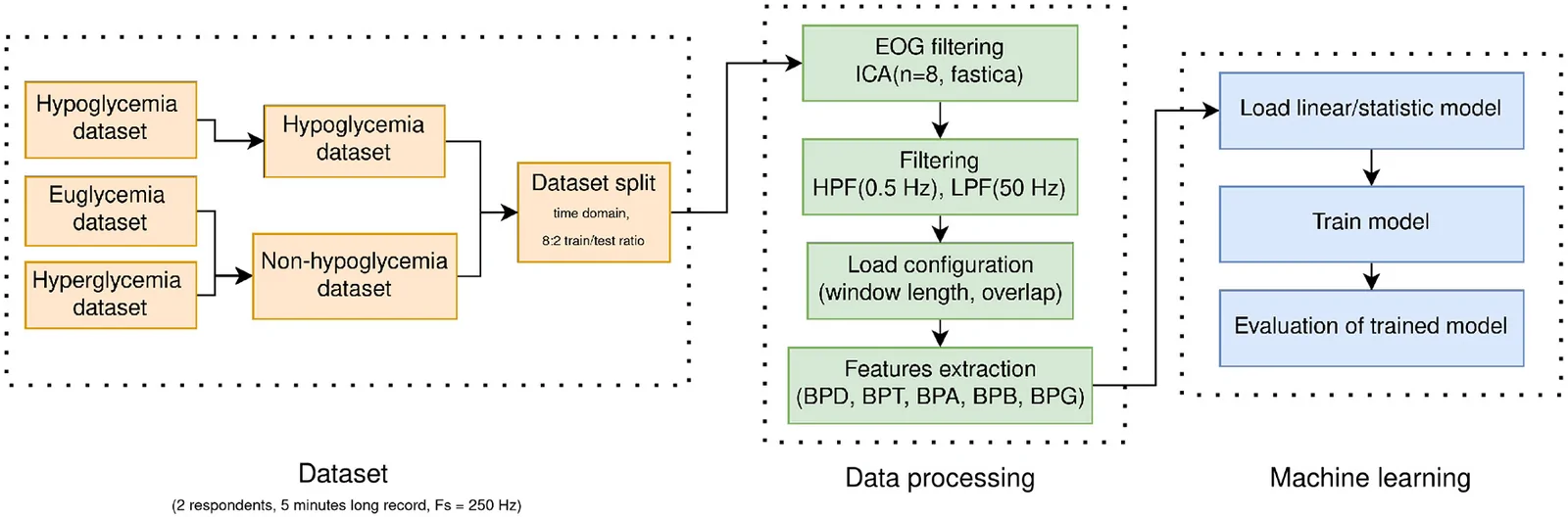
Workflow of the proposed hypoglycemia classification pipeline. EEG recordings acquired from two participants with type 1 diabetes were initially categorized into hypoglycemia, euglycemia, and hyperglycemia classes. For classification, euglycemia and hyperglycemia were merged into a non-hypoglycemia class, resulting in a binary classification dataset. The data were split chronologically into training and testing sets (8:2 ratio), pre-processed, segmented, and transformed into frequency-domain band-power features. Linear and statistical machine learning models were subsequently trained and evaluated across multiple segmentation configurations.

The methodology of this study is uniquely centered on a systematic investigation of how different segmentation granularities and machine learning model complexities interact to influence classification performance within data-limited BCI scenarios. Unlike traditional approaches that may prioritize high-parameter deep learning architectures, our method prioritizes computationally efficient and low-complexity models to specifically evaluate their feasibility for real-time deployment on power-constrained embedded and wearable platforms. Workflow (described on Figure 1) is divided to 3 steps: (i) Creation of dataset, (ii) Data processing and (iii) Machine learning.

This study is a preliminary feasibility investigation based on a very small cohort and is intended to explore whether EEG-based glycemic state discrimination is detectable under controlled conditions, not to establish generalizable performance.

### 3.1 Data acquisition

**Figure 2:**
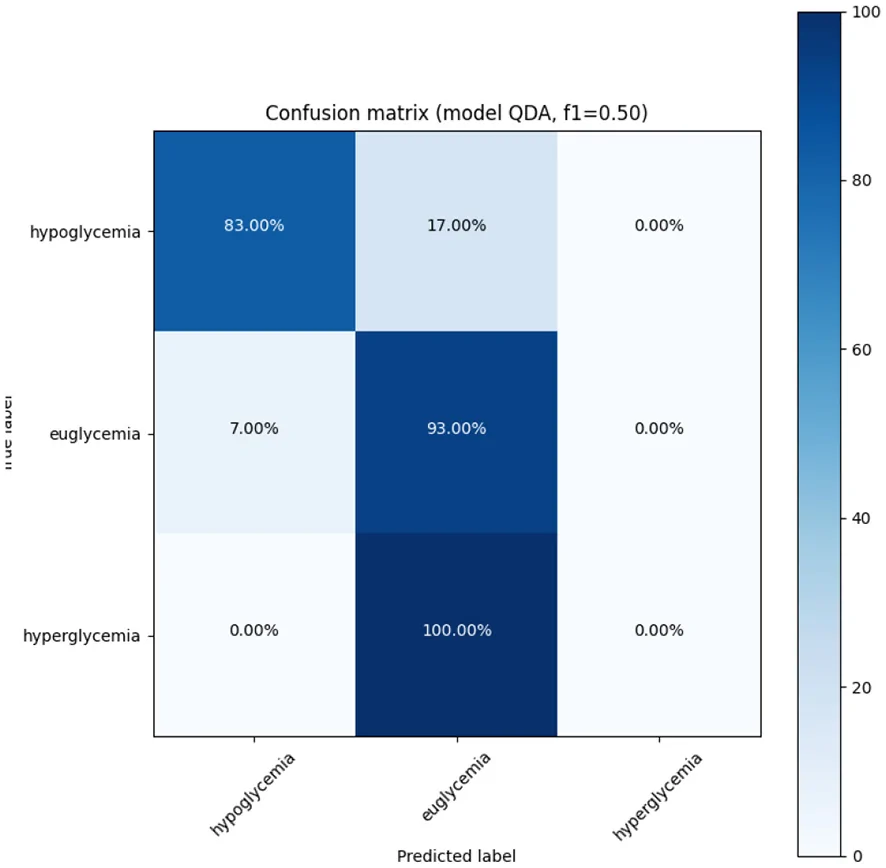
Confusion matrix obtained from a preliminary three-class (hypoglycemia, euglycemia, hyperglycemia) QDA classification using data from T1D patients (window = 15 s, step = 3.75 s, 25% overlap).

EEG data were acquired using the BrainAccess Mini device with sampling frequency *F*_*s*_ = 250Hz. EEG was recorded from eight scalp electrodes positioned according to the international 10-20 system: F3, F4, C3, C4, P3, P4, O1, and O2. These channels were selected to provide coverage of frontal, central, parietal, and occipital cortical regions while remaining compatible with portable EEG acquisition hardware. To support real-time streaming and reliable synchronization with metabolic measurements, the acquisition pipeline was integrated with the Lab Streaming Layer (LSL) framework. In the experiment, two participants diagnosed with T1D were monitored under controlled conditions: one participant used an insulin pump, while the other relied on multiple daily insulin injections. The purpose of the recording protocol was to capture brain activity in clinically relevant glycemic states (hypoglycemia, euglycemia and hyperglycemia) and to allow direct alignment of the EEG segments with continuous glucose monitoring (CGM) data.^1^ For classification purposes, euglycemia and hyperglycemia were merged into a single non-hypoglycemic class. Preliminary three-class experiments (hypoglycemia, euglycemia, hyperglycemia) showed complete overlap between euglycemia and hyperglycemia in the EEG feature space, with hyperglycemia samples consistently classified as euglycemia (Figure 2). Consequently, the analysis focused on the clinically relevant discrimination between hypoglycemia and non-hypoglycemia.

It is acknowledged that the current dataset comprises recordings from two participants with Type 1 diabetes. While this sample size is consistent with early-stage feasibility and proof-of-concept investigations in BCI research, where the primary objective is to establish the existence of a detectable neurophysiological signal rather than to demonstrate population-level generalizability, it constitutes a significant constraint on the statistical power and external validity of the reported findings. The results should therefore be interpreted as preliminary evidence supporting the plausibility of EEG-based glycemic state classification, rather than as definitive performance benchmarks. All reported classification metrics (accuracy, macro-F1) reflect within-subject performance under controlled conditions and are not intended to generalize across individuals or clinical settings at this stage.

In addition to the two participants with T1D, five healthy volunteers were recruited as a baseline comparison group. EEG recordings were acquired using the same BrainAccess Mini device and electrode configuration. Healthy participants were recorded under standardized postprandial conditions, with measurements performed 30 min and 180 min after meal ingestion. The same EEG acquisition and pre-processing procedures were applied to both groups; however, CGM synchronization was performed only for the T1D participants to enable glycemic-state labeling. Healthy controls were included solely for baseline comparison of EEG spectral characteristics.

To facilitate reproducibility, Table 1 summarizes the study cohort, recording protocol, and the data available for subsequent EEG segmentation and classification. In this manuscript, a “recording” refers to a continuous EEG acquisition period, whereas a “window” denotes a segmented EEG epoch generated during pre-processing and used as an individual sample for machine-learning analysis.

**Table 1:** Summary of study participants and EEG acquisition protocol.

Parameter	T1D group	Healthy controls
Participants	2	5
Recording sessions per participant	3	3
Recording duration per session (min)	5	5
Total EEG recordings	6	15
Total recording time (min)	30	75
EEG montage	8 channels (10–20 system)	8 channels (10–20 system)
CGM synchronized	Yes	No

The 30-min recording corresponded to the early postprandial phase following meal ingestion, during which blood glucose levels typically rise and approach peak concentrations (Liu et al., 2022). In individuals with diabetes, this phase is often associated with postprandial glucose excursions and transient hyperglycemia (Davies et al., 2018).

The 180-min recording represented a late postprandial phase characterized by declining glucose levels mediated by insulin action. In both healthy and diabetic populations, this period may be associated with reactive hypoglycemia or relative glucose deficit (Altuntaş, 2019; Li et al., 2023; Wei et al., 2025). Previous studies have shown that hypoglycemia is accompanied by cortical slowing, reflected by increased low-frequency EEG activity (Ryu et al., 2019).

Although the use of fixed postprandial time points does not account for inter-individual variability in glucose kinetics (Bellini et al., 2021; Thomas et al., 2024), the selected protocol provides a standardized and reproducible framework for comparing neurophysiological responses between healthy individuals and participants with T1D.

### 3.2 Signal processing and feature extraction

The EEG processing pipeline was designed with low computational complexity to support future deployment on embedded platforms. The analysis consisted of splitting dataset in time domain, pre-processing, segmentation, and feature extraction performed in the frequency domain.

The EEG processing workflow consisted of the following sequential steps:

1. Raw EEG acquisition from eight scalp electrodes (F3, F4, C3, C4, P3, P4, O1, and O2) at a sampling frequency of 250 Hz.
2. Assignment of glycemic-state labels using synchronized CGM measurements. For classification, euglycemic and hyperglycemic samples were merged into a single non-hypoglycemia class.
3. Exclusion of the first 2 min of each recording to eliminate electrode-settling and initialization effects.
4. Chronological train-test partitioning of each recording using an 80:20 split.
5. EEG pre-processing using ICA-based ocular artifact removal and 0.5–50 Hz band-pass filtering.
6. Segmentation of recordings into analysis windows using multiple window lengths *W*∈{5, 6, 8, 10, 12, 15, 20, 22, 25, 28, 30, 45} s and relative step sizes {0.5, 0.75, 0.8, 0.9, 1.0}.
7. Extraction of relative δ-, θ-, α-, β-, and γ-band powers for each channel and each window.
8. Training of a QDA classifier for each segmentation configuration.
9. Evaluation of each configuration using accuracy and macro-F1 score.
10. Selection of the best-performing configurations and analysis of the associated confusion matrices.

#### 3.2.1 Dataset split

Across all classes, the dataset was partitioned into training and test sets using an 80:20 split. To mitigate the potential misclassification arising from temporal overlap in time–frequency representations, the split was performed chronologically within each recording: the first 80% of each record was assigned to the training set and the remaining 20% to the test set.

Cross-validation across participants was not performed in this study because the dataset comprised only two individuals with type 1 diabetes. With such a limited cohort, subject-level cross-validation approaches (e.g., leave-one-subject-out or k-fold cross-validation across subjects) would not provide meaningful estimates of generalization and could be misleading. All reported results should therefore be interpreted as within-subject, pilot-level feasibility rather than population-level validation.

#### 3.2.2 Pre-processing

Although the recordings were conducted under controlled resting-state conditions with participants seated and minimizing movement, standard artifact mitigation procedures were applied to ensure signal integrity. Raw EEG signals were filtered using a band-pass filter in the range 0.5 Hz ≤ *f* ≤ 50 Hz, which retains the dominant clinical EEG bands (delta, theta, alpha, beta, and gamma) while attenuating slow baseline drift, high-frequency noise, and muscular artifacts that predominantly occur above 30 Hz. The 50 Hz upper cutoff of the bandpass filter restricts gamma-band analysis to the lower portion of the gamma range (30–45 Hz), as the filter transition band attenuates frequencies approaching 50 Hz. As the recording environment operates at 50 Hz mains frequency, a notch filter was not separately applied; however, this constraint is acknowledged as potentially affecting the reliability of gamma-band power estimates. Given that the primary discriminative features identified in this study involve delta and beta bands, this limitation does not substantially affect the core findings.

Automated independent component analysis (ICA) has been used for EOG artifacts removal. However, given the limited number of EEG channels in the BrainAccess Mini device, ICA decomposition may be suboptimal and could lead to partial removal of meaningful neural signals along with ocular artifacts. EMG-related artifacts were not specifically removed because dedicated electromyographic recordings were not available. Consequently, residual muscle activity may remain present in the EEG signals, particularly in the beta and lower gamma frequency ranges. To eliminate transient non-physiological disturbances associated with electrode settling, device initialization, and initial calibration, the first 2 min of each recording were excluded from further processing. The controlled experimental protocol, including the absence of motor tasks, speech, or ambulatory activity, inherently reduced the overall artifact burden, making the applied pre-processing pipeline sufficient for this feasibility study.

#### 3.2.3 Time–frequency analysis and band power estimation

After pre-processing, the EEG was analyzed using short-time windowing to capture time-varying spectral content while maintaining computational efficiency. Band power features were computed through a time–frequency approach using a sliding window.

We evaluated a range of temporal window lengths *w*∈{5, 6, 8, 10, 12, 15, 20, 22, 25, 28, 30, 45} to assess the sensitivity of the analysis to segmentation granularity. For each window length, the sliding step was defined as a fraction of the window size, using {0.5, 0.75, 0.8, 0.9, 1.0}, thereby spanning configurations from overlapping to non-overlapping windows. Expressing the overlap as a relative ratio, rather than as a fixed number of samples, ensures that the proportion of shared data between adjacent segments remains constant across different window lengths. This prevents window-size–dependent bias in the effective overlap and avoids unequal redundancy levels that could otherwise influence classification accuracy and confound the interpretation of segmentation effects. All combinations of window length and relative step were tested under otherwise identical pre-processing and training conditions.

The number of analysis windows depended on the selected window length and overlap configuration. Consequently, no single window count applies to all experiments. For a recording duration of 5 min, non-overlapping segmentation generated between approximately 6 windows (*W* = 45 s) and 60 windows (*W* = 5 s) per recording across the evaluated settings. For the best-performing configuration (*W* = 6 s, *S* = 6 s, 0% overlap), each recording yielded approximately 50 windows.

For each channel and window, the power spectral density was estimated and summarized as relative band power values for the delta, theta, alpha, beta, and gamma bands. This representation provides a compact descriptor of brain activity dynamics and is suitable for detecting state-dependent changes associated with glucose fluctuations. Relative band power is among the most widely used feature sets in electroencephalography-based classification due to its low computational complexity, interpretability, and robust performance reported in prior studies. The present work is conceived as a feasibility study demonstrating that clinically relevant glycemic states can be identified using computationally efficient features; future research may incorporate additional feature types to further enhance discriminative performance. No additional feature normalization was applied because relative band-power features are inherently normalized with respect to the total spectral power within each window.

Class labels were assigned at the window level using synchronized CGM measurements. Windows corresponding to glucose values below the hypoglycemia threshold were assigned to the hypoglycemia class, whereas euglycemic and hyperglycemic windows were merged into the non-hypoglycemia class.

#### 3.2.4 Pre-processing, window description

To mitigate temporal autocorrelation between adjacent training and test samples, the primary classification results are reported for non-overlapping windows (step = window size, i.e., 0% overlap). Overlapping configurations were additionally evaluated to assess whether inflated accuracy occurs at higher overlap ratios; these results are reported in Section 4.1.

### 3.3 Results of pre-processing and exploratory analysis

The pre-processing and feature extraction pipeline revealed consistent band power patterns associated with clinically relevant glucose levels, supporting the feasibility of EEG-derived biomarkers for the detection of hypoglycemia and hyperglycemia. The results reported in this section were obtained using a window length of 10s with a relative sliding step of 0.5 (i.e., 50% overlap). Importantly, synchronizing EEG streams with CGM measurements enabled a direct comparison between neurophysiological activity and glucose dynamics, which is essential for designing predictive monitoring systems, particularly for individuals with impaired hypoglycemia awareness.

**Figure 3:**
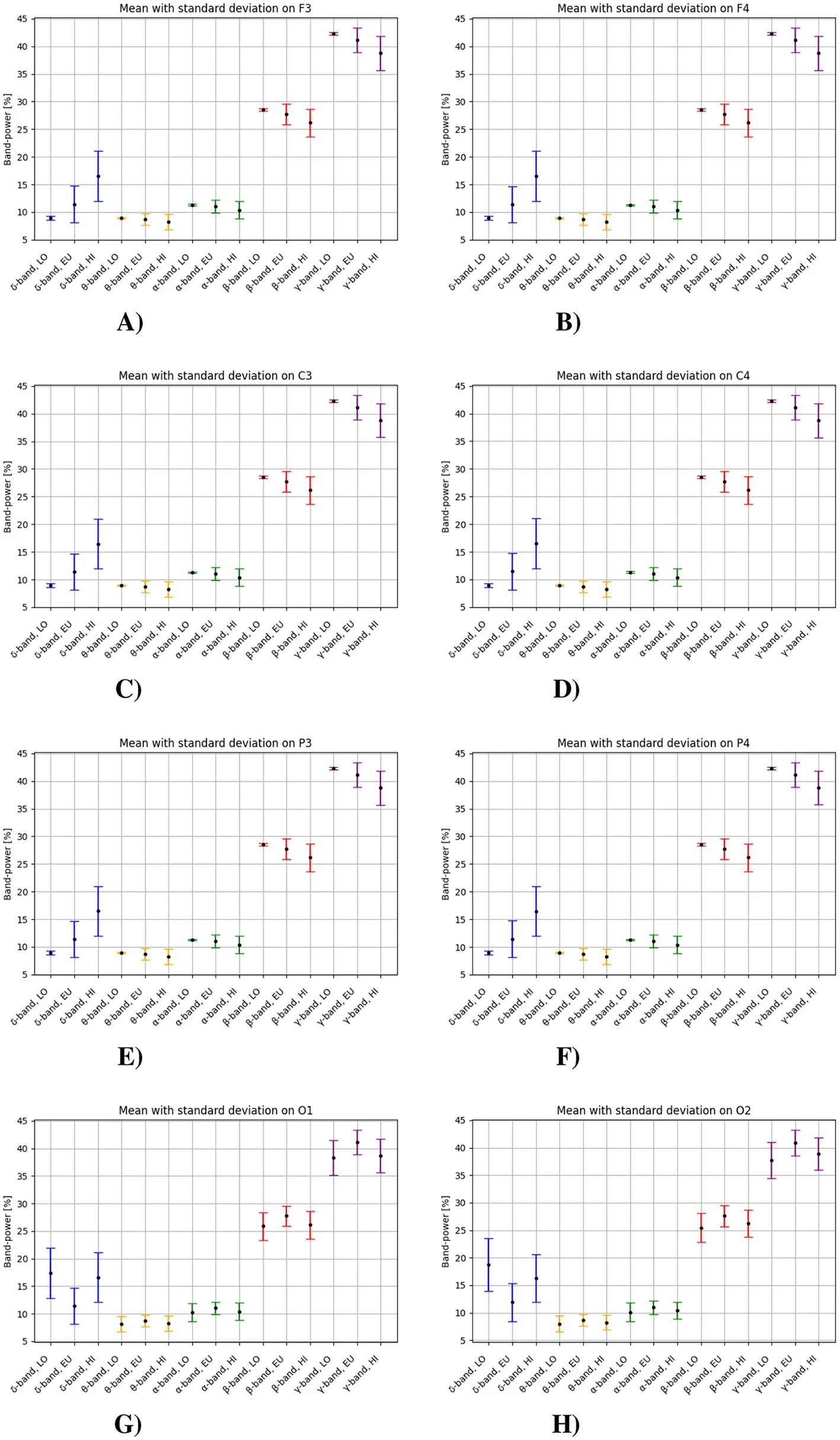
Results of the band powers across electrodes, using 10—20 system. **(A)** Electrode F3, **(B)** Electrode F4, **(C)** Electrode C3, **(D)** Electrode C4, **(E)** Electrode P3, **(F)** Electrode P4, **(G)** Electrode O1, **(H)** Electrode O2. (LO, hypoglycemia; EU, euglycemia; HI, hyperglycemia; two T1D patients included).

Across electrodes, the most pronounced differences associated with hypoglycemia were reflected in the mean and standard deviation of delta- and beta-band power, particularly at electrodes F4, C4, and P4. With increasing blood glucose level (from hypoglycemia to hyperglycemia), delta-band power increased, whereas beta-band power decreased, while the remaining bands exhibited only minor changes. For visual comparison of these trends across glycemic states, Figures 3A–H present band power trajectories for each electrode.

### 3.4 Comparison of T1D and healthy subjects under postprandial conditions

To provide a baseline reference for EEG spectral activity, relative band power was compared between healthy controls (*n* = 5) and participants with T1D (*n* = 2) under standardized postprandial conditions (Table 2).

**Table 2:** Relative EEG band power comparison based on measured data during hyperglycemic (30 min) and hypoglycemic (180 min) conditions.

Electrode	Group	30 min					180 min				
		δ-band	θ-band	α-band	β-band	γ-band	δ-band	θ-band	α-band	β-band	γ-band
F3	Healthy	0.18	0.17	0.27	0.23	0.14	0.19	0.23	0.22	0.22	0.14
	T1D	0.27	0.19	0.23	0.19	0.11	0.23	0.29	0.17	0.19	0.13
F4	Healthy	0.19	0.16	0.26	0.22	0.15	0.20	0.22	0.21	0.23	0.14
	T1D	0.31	0.18	0.22	0.17	0.12	0.24	0.27	0.18	0.19	0.13
C3	Healthy	0.17	0.19	0.28	0.21	0.15	0.18	0.25	0.20	0.23	0.14
	T1D	0.28	0.22	0.22	0.18	0.11	0.22	0.30	0.16	0.20	0.12
C4	Healthy	0.18	0.17	0.26	0.23	0.14	0.19	0.24	0.20	0.22	0.15
	T1D	0.30	0.19	0.21	0.18	0.12	0.23	0.28	0.17	0.20	0.13
P3	Healthy	0.17	0.20	0.29	0.20	0.14	0.18	0.26	0.21	0.22	0.13
	T1D	0.26	0.23	0.22	0.18	0.11	0.21	0.31	0.16	0.19	0.13
P4	Healthy	0.18	0.18	0.27	0.22	0.15	0.19	0.24	0.21	0.22	0.14
	T1D	0.29	0.20	0.22	0.18	0.12	0.22	0.29	0.17	0.20	0.12
O1	Healthy	0.16	0.18	0.30	0.21	0.15	0.17	0.27	0.21	0.22	0.13
	T1D	0.25	0.21	0.24	0.18	0.12	0.20	0.30	0.17	0.20	0.13
O2	Healthy	0.17	0.17	0.29	0.22	0.15	0.18	0.25	0.22	0.22	0.13
	T1D	0.27	0.20	0.23	0.18	0.12	0.21	0.29	0.17	0.20	0.13

Across most electrodes and recording conditions, participants with T1D exhibited higher relative delta-band power and lower alpha- and beta-band power than healthy controls. These differences were consistently observed in frontal, central, parietal, and occipital regions.

The observed trends are consistent with the hypothesis that glucose dysregulation is associated with measurable alterations in EEG spectral composition. Although the limited sample size does not permit formal statistical inference, the comparison provides physiological context for the EEG features identified in the classification analysis and supports the use of healthy controls as a baseline reference group.

## 4 Results

This section presents a comparative evaluation of methodological approaches for classifying hypoglycemia vs. non-hypoglycemia from EEG recordings, using data-efficient approaches based on classical machine learning models, which can perform reliably even when training data are scarce. These experiments enable a systematic comparison of performance and practical feasibility, highlighting the trade-offs between conventional methods that require limited data and modern deep learning approaches that benefit from augmented training sets.

**Figure 4:**
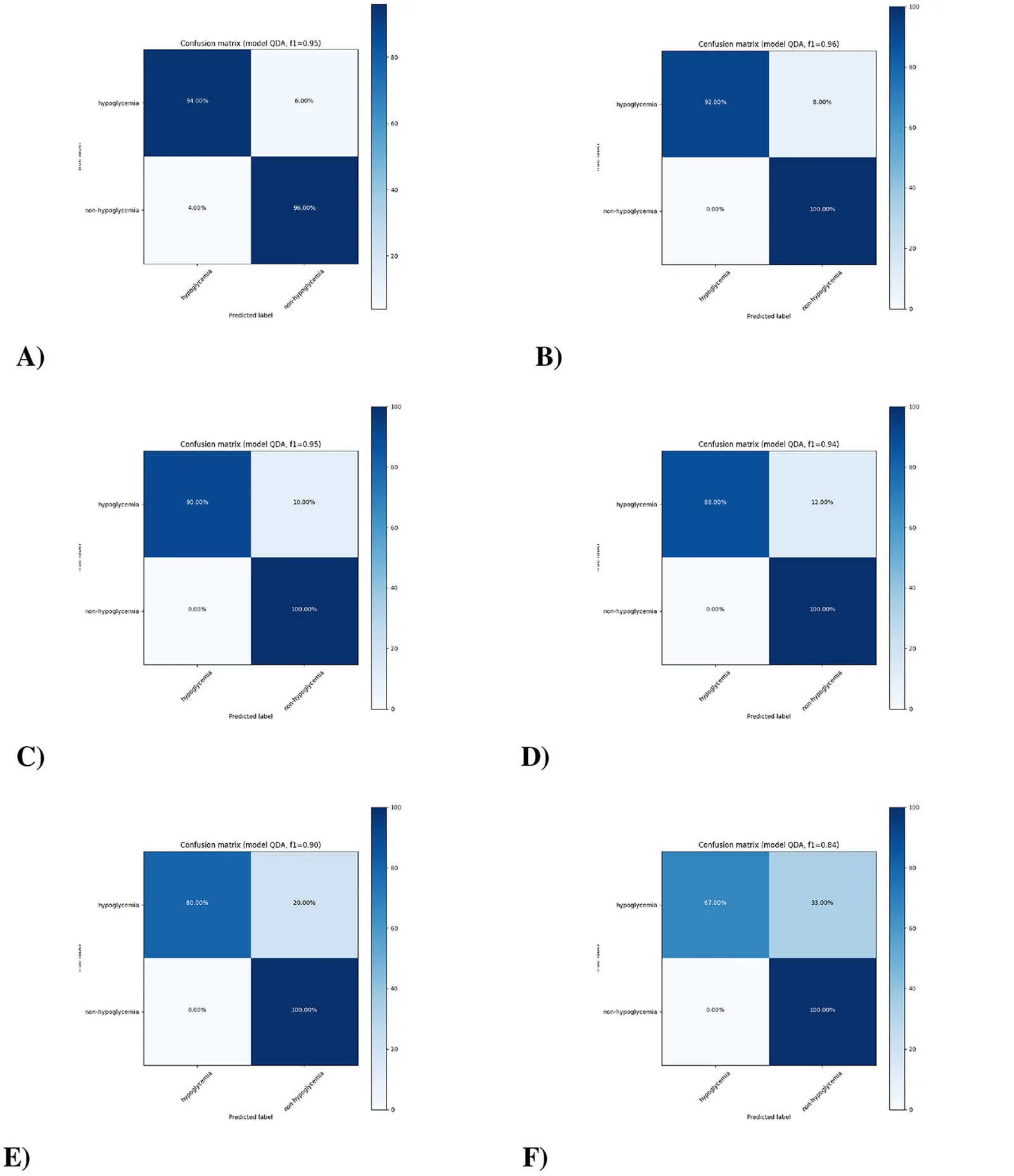
Confusion matrices with best results with defined window size (W), step size (S), and overlap. **(A)** QDA, *W* = 5 s, *S* = 5 s (0% overlap. **(B)** QDA, *W* = 6 s, *S* = 6 s (0% overlap). **(C)** QDA, *W* = 8 s, *S* = 8 s (0% overlap). **(D)** QDA, *W* = 10 s, *S* = 10 s (0% overlap). **(E)** QDA, *W* = 15 s, *S* = 15 s (0% overlap). **(F)** QDA, *W* = 22 s, *S* = 22 s (0% overlap).

In addition to overall accuracy and macro-F1, we examined class-specific performance for the hypoglycemia class, as this represents the most clinically critical condition. The confusion matrices (Figures 4A–F) show that the majority of hypoglycemic events were correctly identified, indicating a high sensitivity for hypoglycemia. Misclassifications primarily involved hypoglycemia being predicted as non-hypoglycemia, with the opposite error occurring less frequently across all configurations. Given the clinical importance of hypoglycemia detection, we report sensitivity and emphasize that most errors correspond to missed hypoglycemic events.

The strong performance of QDA is consistent with prior studies demonstrating the effectiveness of discriminant analysis classifiers in EEG-based mental state and physiological event classification under limited data conditions (Aditya and Tibarewala, 2012; Dos Santos et al., 2023).

### 4.1 Effect of segmentation parameters

EEG signals were segmented using multiple combinations of window length *W* and step size *S*, both expressed in seconds. All recordings were sampled at a fixed sampling frequency of *f*_*s*_ = 250 Hz. For each segmentation configuration, the QDA model has beem tested. Table 3 reports the mean classification accuracy (*acc*_*mean*_), and the mean F1-score (*F*1_*macro*_) computed across repeated runs.

**Table 3:** Model and classification performance for each segmentation configuration (*f*_*s*_ = 250 Hz), with 0% window overlap.

*W* [s]	Model	*acc*_*mean*_	Mean *F*1_*macro*_
5	QDA	0.95000	0.94999
6	QDA	0.96212	0.96201
8	QDA	0.94949	0.94941
10	QDA	0.93750	0.93726
12	QDA	0.92063	0.91933
15	QDA	0.90196	0.90058
20	QDA	0.87500	0.87302
22	QDA	0.84848	0.83902
25	QDA	0.83333	0.82857
28	QDA	0.78260	0.75269
30	QDA	0.56522	0.36111
45	QDA	0.61538	0.38095

To examine whether temporal autocorrelation between overlapping windows inflates classification performance, Table 4 reports accuracy and macro-F1 for varying overlap ratios at the best-performing window length (W = 6s).

**Table 4:** Effect of window overlap on classification accuracy (*W* = 6 s, QDA).

*W* [s]	*S* [s]	Relative step	Overlap [%]	*acc*_*mean*_	*F*1_*macro*_
6	6.0	1.00	0	0.96212	0.96201
6	5.4	0.90	10	0.95139	0.95127
6	4.8	0.80	20	0.95706	0.95700
6	4.5	0.75	25	0.82759	0.82612
6	3.0	0.50	50	0.97276	0.97272

A potential confound in EEG window-based classification is temporal autocorrelation between adjacent overlapping segments, which can inflate accuracy estimates when training and test windows are temporally proximate. To address this, classification performance was compared across overlap levels at a fixed window length of W = 6s (Table 4). The stable trend observed as overlap increases confirms that the reported performance is not substantially driven by temporal redundancy between adjacent segments. Given that primary results are reported at 0% overlap, where no shared data exists between adjacent windows and performance remains consistent across higher overlap ratios, the core findings are robust with respect to this concern.

Overall, the results indicate that segmentation strongly influences classification performance. Short windows (approximately 5–10 s) yield the highest accuracy and F1 scores, while performance steadily declines as window length increases. This trend suggests that longer windows reduce the effective sample count and introduce excessive temporal smoothing, which degrades classification performance in data-limited settings. In all configurations, QDA consistently achieved the best results. Peak performance was observed at *W* = 6 s, with *acc*_*mean*_ = 0.96212 and *F*1_*macro*_ = 0.96201, followed closely by similarly strong results at *W* = 5 s and *W* = 8 s. In contrast, performance drops substantially for window lengths beyond 25–30 s, confirming that shorter segments provide more reliable and discriminative representations for the task.

To further evaluate the statistical significance of the segmentation effect, a Wilcoxon signed-rank test was performed between the best and worst performing configurations reported in Table 3. Specifically, the configuration with *W* = 6 s (*F*1_*macro*_ = 0.96201) was compared with the lowest-performing configuration *W* = 45 s (*F*1_*macro*_ = 0.38095). The test revealed a statistically significant difference, with the Wilcoxon statistic reaching the minimum possible value (*W* = 0.0) and a p-value of 1.907 × 10^−6^. This indicates that the best configuration consistently outperformed the worst configuration across the evaluated runs, confirming that the choice of segmentation parameters has a substantial impact on the classification performance. It is important to note that this statistical comparison evaluates the consistency of segmentation effects across repeated classification runs, not across independent participants. Subject-level generalizability cannot be assessed from the present dataset.

### 4.2 Confusion matrices for models with best *f*_1_ macro

To provide a qualitative view of model behavior, we report confusion matrices for representative binary classification settings distinguishing hypoglycemia from non-hypoglycemia (Figures 4A–F). These matrices enable comparison of error patterns across configurations, highlighting both overall performance and class-specific misclassifications. Such analysis is critical for clinical evaluation, particularly to determine whether hypoglycemic events are misclassified as non-hypoglycemia.

## 5 Discussion

The results of this study demonstrate that hypoglycemia can be effectively classified against non-hypoglycemia using non-invasive EEG signals and computationally efficient feature representations. The observed separability between these classes, based on band-power features, suggests that glucose-related metabolic disturbances are reflected in measurable neurophysiological changes. Notably, the consistent involvement of delta- and beta-band power across frontal, central, and parietal regions highlights their potential as biomarkers for binary glycemic state classification.

The evaluation of multiple segmentation configurations highlights the importance of window length in EEG-based classification tasks. The results indicate that medium window durations combined with moderate overlap provide a favorable trade-off between temporal resolution, feature stability, and classification performance. These findings are especially relevant for real-time applications, as segmentation parameters directly influence computational load and latency. The strong performance of classical data-efficient classifiers, particularly Quadratic Discriminant Analysis (QDA), further suggests that reliable classification can be achieved without resorting to complex or resource-intensive models, which is advantageous for deployment on low-power, battery-operated devices.

From a clinical perspective, false negatives in hypoglycemia detection are of particular concern, as missed hypoglycemic events can lead to severe neurological consequences. The confusion matrices indicate that, while hypoglycemia is detected with high specificity, a non-negligible portion of hypoglycemic samples is misclassified as non-hypoglycemia, directly corresponding to this critical error type. This pattern highlights the importance of prioritizing sensitivity when evaluating model performance, as overall accuracy alone may obscure clinically relevant shortcomings. In line with broader efforts to develop non-invasive alternatives to direct glucose measurement, this study does not aim to estimate blood glucose levels explicitly but instead focuses on the detection of hypoglycemic events as a clinically meaningful and computationally tractable objective. The observed performance demonstrates the potential of EEG-based systems as complementary monitoring tools for the identification of hazardous hypoglycemic states, particularly in individuals with impaired hypoglycemia awareness.

A comparison with prior literature reveals both consistencies and discrepancies in the electrophysiological signatures of glycemic disturbances. Ngo et al. (2021) reported increased theta activity and reduced alpha power during hypoglycemia, and elevated high-frequency power during hyperglycemia, with limited changes in delta and beta bands. In contrast, our findings highlight delta- and beta-band involvement. These differences may stem from experimental conditions and methodology: Ngo et al. conducted nighttime recordings, where sleep-related dynamics can modulate spectral content, whereas our data were collected during wakefulness. Additionally, their use of spectral moments differs from our relative band-power approach, emphasizing different characteristics of the EEG signal. Despite these discrepancies, the observed increase in low-frequency activity during hypoglycemia remains consistent with cortical slowing under neuroglycopenia, suggesting that EEG correlates of glycemic states are strongly context-dependent.

Our findings are also broadly consistent with previous reports describing hypoglycemia-associated EEG slowing and increased low-frequency activity during periods of glucose deprivation (Blaabjerg and Juhl, 2016; Ryu et al., 2019). Sejling et al. (2017) similarly observed reproducible EEG alterations during hypoglycemia in individuals with T1D, supporting the robustness of neurophysiological responses to glucose dysregulation. Although the specific spectral features identified across studies differ, these variations are likely influenced by differences in participant populations, recording conditions, and feature extraction methodologies. Nevertheless, the collective evidence supports the presence of measurable and reproducible EEG alterations during hypoglycemia, reinforcing the feasibility of EEG-based monitoring approaches for detecting clinically relevant glycemic disturbances. Furthermore, the successful detection of hypoglycemia using alternative EEG modalities, such as ear-EEG (Cabrera et al., 2024), highlights the broader potential of non-invasive neurophysiological monitoring systems for future diabetes management applications.

In addition to the classification analysis, comparison with the healthy control group provided a baseline reference for EEG spectral activity. Across multiple electrodes, participants with T1D exhibited higher relative delta-band power and lower alpha- and beta-band power than healthy controls under both recording conditions. Although the limited sample size precludes formal statistical inference, these observations support the presence of diabetes-related alterations in EEG spectral composition and provide physiological context for the classification results.

An additional limitation concerns the potential contribution of EMG activity to the recorded EEG signals. Although recordings were acquired under controlled resting-state conditions and participants were instructed to minimize movement, scalp EEG electrodes may also capture activity originating from facial, scalp, and neck muscles. This issue is particularly relevant during hypoglycemia, which may be accompanied by tremor or increased muscle tension. Because dedicated EMG channels were not recorded, EMG contamination could not be explicitly quantified or removed, and therefore part of the observed beta-band differences may reflect a combination of neural and muscular activity. Future studies should incorporate dedicated EMG recordings or EMG-sensitive artifact rejection methods to further validate the specificity of the identified EEG features.

Deep learning architectures were not included in the present comparison, as the limited dataset size (two subjects, 5 min per class) precludes reliable DL training without severe overfitting risk. Moreover, the deployment objective of this work, which is real-time, embedded monitoring on low-power wearable devices favors computationally lightweight classical models over resource-intensive neural networks.

The most consequential limitation of this study is the small number of participants (n = 2). From a statistical standpoint, performance estimates derived from two subjects carry wide confidence intervals and are highly susceptible to individual variability in EEG characteristics, glycemic response patterns, and inter-session signal stationarity. The observed classification performance while promising may reflect subject-specific spectral patterns rather than a generalized neurophysiological marker of glycemic state. Consequently, the reported accuracy and F1-scores should not be interpreted as population-representative benchmarks. No cross-subject generalization analysis was possible at this sample size, and leave-one-subject-out or k-fold cross-validation across subjects which is a standard practice for demonstrating model robustness in BCI research, could not be meaningfully applied.

Because the sample includes only two T1D subjects, we did not perform cross-subject cross-validation. The study is therefore explicitly positioned as a pilot proof-of-concept, and future work must involve larger cohorts and subject-level cross-validation to assess generalizability.

These statistical constraints are inherent to the early-stage, data-limited nature of the study and are explicitly acknowledged as a primary motivation for the planned cohort expansion.

## 6 Conclusion

This study investigated the feasibility of classifying diabetes-related critical physiological events using non-invasive EEG recordings, with a particular focus on hypoglycemia in individuals with type 1 diabetes. Baseline EEG characteristics were additionally compared with recordings from healthy controls to provide physiological context for the observed T1D-related spectral patterns. Building on prior findings that associate glucose dysregulation with characteristic EEG spectral changes, the proposed approach integrates low-complexity signal processing with data-efficient machine learning methods to enable real-time monitoring scenarios.

EEG data were acquired using a portable BCI device and synchronized with continuous glucose monitoring through the Lab Streaming Layer framework, enabling direct alignment between neurophysiological activity and glycemic states. A computationally efficient pre-processing and feature extraction pipeline was designed, relying on band-limited filtering and time–frequency analysis of EEG band power. Exploratory analysis revealed consistent trends across subjects and electrodes, with hypoglycemia associated primarily with changes in delta-band and beta-band power, particularly over frontal, central, and parietal regions. These observations are consistent with physiological findings reported in the literature and support the use of EEG spectral features as biomarkers of glucose-related brain activity.

For event classification, multiple segmentation strategies were evaluated to assess the influence of window length and overlap on performance. Classical machine learning models, particularly Quadratic Discriminant Analysis demonstrated preliminary performance under constrained experimental conditions across a wide range of configurations, achieving high accuracy and macro-averaged F1-scores even under limited data availability. Confusion matrix analysis further confirmed that appropriate segmentation can substantially reduce clinically relevant misclassifications.

Overall, the results demonstrate that EEG-based discrimination between hypoglycemia and non-hypoglycemia is feasible using non-invasive sensors and computationally lightweight algorithms. The combination of spectral EEG features, optimized segmentation and classical classifiers offers a promising foundation for future closed-loop or embedded implementations aimed at early detection and mitigation of hypoglycemia, particularly in patients with impaired hypoglycemia awareness.

Because this study enrolled only two T1D participants and five controls, it should be viewed as a proof-of concept rather than a definitive validation of EEG-based hypoglycemia detection. In future work, we will (i) recruit a larger T1D cohort to obtain sufficient statistical power, (ii) apply subject-level cross-validation (e.g., leave-one-subject-out) to evaluate generalizability, and (iii) systematically document recordings, trials, and window counts for full reproducibility. These extensions are essential to move from exploratory findings to a clinically reliable, deployable BCI system.

Future work will focus on expanding the subject cohort, increasing the amount of available data per participant through longitudinal recordings, evaluating adaptive and personalized models, and applying dataset augmentation strategies to improve model generalization, inter-subject robustness, long-term feature stability, and clinical applicability. Importantly, the present recordings were obtained under controlled conditions in which participants were in a relaxed state without performing specific physical or cognitive tasks. Future measurements will therefore include recordings acquired during daily activities in order to assess robustness under more realistic conditions and to evaluate the influence of movement- and workload-related artifacts. In parallel, we plan to implement and evaluate deep neural network architectures capable of learning hierarchical representations directly from time–frequency features or minimally processed signals, potentially improving performance relative to conventional classifiers. At the same time, the current results demonstrate that even computationally simple and data-efficient models are feasible for hypoglycemia classification, supporting their use as complementary safety mechanisms alongside existing glucose monitoring technologies. Additionally, the integration of multimodal physiological signals, such as electrocardiography and heart rate–related measures, may further enhance classification robustness by capturing complementary aspects of autonomic and metabolic regulation. Such extensions will enable investigation of cross-modal feature interactions while maintaining a non-invasive sensing paradigm, ultimately supporting the development of practical, real-time systems for safer management of diabetes-related critical events.

Several directions for future research are identified to build upon the preliminary findings of this study. The most immediate priority is the expansion of the participant cohort to a statistically sufficient sample size. A minimum of 15–20 subjects to ensure adequate statistical power, thus reducing the influence of inter-individual variability, and that will enable robust estimation of confidence intervals around classification metrics. A larger cohort will further allow the application of rigorous cross-subject validation strategies including leave-one-subject-out (LOSO) evaluation and stratified k-fold cross-validation which are considered standard practice for demonstrating model generalizability in EEG-based BCI research. In parallel, longitudinal recordings spanning multiple sessions per participant will be pursued to assess the temporal stability of the identified spectral biomarkers and to evaluate classifier robustness against intra-subject signal drift over time. Deep learning architectures, including convolutional and recurrent neural networks, will be systematically evaluated once sufficient training data are available, enabling a rigorous comparison against the classical models reported here. Recordings acquired during daily-life activities rather than controlled resting-state conditions will also be incorporated to assess the robustness of the proposed pipeline under realistic ambulatory conditions. Collectively, these extensions aim to transition the current work from a proof-of-concept demonstration to a clinically validated and deployable system for early, non-invasive detection of critical glycemic events in individuals with type 1 diabetes.

## Data Availability

The raw data supporting the conclusions of this article will be made available by the authors, without undue reservation.

## Footnotes

1 This study has been conducted in accordance with the ethical standards of the University of Žilina, Approval r.n.: EK UNIZA 1/2025.
